# Frequency and determinants of phytotherapy use in patients with type 2 diabetes in the Dschang Health District, Cameroon: a cross-sectional study

**DOI:** 10.11604/pamj.2024.47.174.41677

**Published:** 2024-04-09

**Authors:** Michelle Carolle Dongmo Demanou, Sylvain Raoul Simeni Njonnou, André Arsène Bita Fouda, Eric Balti, Fernando Kemta Lekpa, Christian Ngongang Ouankou, Martine Claude Etoa Etoga, Cédric Fritz Gerald Eyenga Bangbang, Marie-Josiane Ntsama Essomba, Anne Ongmeb Boli, Fabrice Lekeufack, Dieudonné Désiré Michel Adiogo

**Affiliations:** 1 Department of Public Health, Faculty of Medicine and Pharmaceutical Sciences, Douala University, Douala, Cameroon,; 2 Department of Internal Medicine and Specialties, Faculty of Medicine and Sciences Pharmaceutical of the University of Dschang, Dschang, Cameroon,; 3 Dschang Regional Hospital Annex, Dschang, Cameroon,; 4 Yaounde Central Hospital, Yaounde, Cameroon,; 5 Diabetes Research Center and Department of Internal Medicine, Universiteit Ziekenhuis Brussel, Vrije Universiteit Brussel, Brussels, Belgium,; 6 Douala General Hospital, Douala, Cameroon,; 7 Yaounde Teaching Hospital, Yaounde, Cameroon,; 8 Department of Internal Medicine and Specialties, Faculty of Medicine and Biomedical Sciences, University of Yaounde I, Yaounde, Cameroon,; 9 Department of Clinical Sciences, Faculty of Health Sciences, University of Bamenda, Bamenda, Cameroon,; 10 Department of Public Health, Faculty of Health Sciences, University of Bamenda, Bamenda, Cameroon,; 11 Department of Biological Sciences of the University of Douala, Faculty of Medicine and Pharmaceutical Sciences of the University of Douala, Douala, Cameroon

**Keywords:** Phytotherapy consumption, type 2 diabetes, risk factors, Dschang, Cameroon

## Abstract

**Introduction:**

phytotherapy is widely used in Africa for the management of many diseases. Data on the use of phytotherapy in people with type 2 diabetes are scarce. We aimed to determine the frequency and factors associated with the consumption/use of phytotherapy products among patients with type 2 diabetes in the Dschang Health District.

**Methods:**

we conducted a cross-sectional study from January to May 2022, including community-dwelling or hospitalized patients with type 2 diabetes who had lived in the Dschang Health District for at least one year. Informed consent was obtained from all patients. Data were collected using a pre-designed questionnaire. Variables collected included socio-demographic characteristics, diabetes knowledge and practices, and perceptions of care.

**Results:**

we included 403 (249 women) patients with type 2 diabetes with a mean (SD) age of 63 (± 14.86) years). Among them, 240 (59.55%) used phytotherapy, either in combination with conventional treatment (168 (41.69%) participants) or not (72 (17.86%) participants), to treat diabetes. The most common reasons for using phytotherapy were easy accessibility and belief in its efficacy. Most patients used both treatments because they thought the combination was more effective. In univariable analysis, we observed a statistically significant association between level of education (p=0.003), socioeconomic level (p<0.001), place of residence (p=0.003), duration of diabetes (p=0.007), and use of phytotherapy. In multivariable analysis, only age between 51 and 60 years (OR: 0.50, 95% CI 0.298 - 0.8521; p=0.01) was associated with the use of phytotherapy.

**Conclusion:**

people living with T2D in the Dschang Health District frequently use phytotherapy as an antidiabetic remedy, especially those aged between 51 and 60 years, those with low education level, low socioeconomic level and medium duration of diabetes. There is a need to evaluate its effectiveness in treating diabetes and its adverse effects.

## Introduction

Diabetes is a chronic metabolic disease characterized by elevated blood glucose (or blood sugar) levels, which over time leads to serious damage to the heart, blood vessels, eyes, kidneys, and nerves. It is recognized as a major cause of premature mortality and disability due to serious complications that can be both acute and chronic [1,2].

Worldwide, the prevalence of diabetes is estimated to be around 10% of the world's population, with 537 million people affected by diabetes in 2021, and the number of people affected is expected to increase in the coming years to 643 million in 2030 and 783 million in 2045. In addition, 6.7 million deaths are attributed to diabetes [2]. Despite advances in the management of type 2 diabetes mellitus (T2DM), treatment goals are often not achieved. Patients who are dissatisfied with the results of conventional medicine often turn to all or part of traditional medicine [3]. Traditional medicine is defined as “the total of knowledge, skills, and practices based on the theories, beliefs, and experiences of different cultures, whether explicable or not, which are used in the maintenance of health and the prevention, diagnosis, alleviation or treatment of physical or mental illness”. One of the main components of traditional medicine is phytotherapy (or herbal medicine), which is the use of medicines derived from plants or herbs to treat or prevent health conditions [4]. A 2018 Canadian study found that 44% of patients used traditional medicines in combination with their conventional treatment [3]. Despite improvements in diabetes guidelines and care, patients in Western countries still use phytotherapy to treat diabetes [5-7].

In Africa, more than 24 million people will be living with diabetes in 2021, and approximately 416,000 deaths will be attributed to diabetes in 2021 [2]. Herbal medicine is used by 80% of the local population for primary health care and there is 1 healer per 500 people compared to 1 doctor per 40,000 people [4]. In rural areas, healers remain the healthcare providers for millions of people [4,8]. Studies carried out in 2015 and 2018 in Morocco and Algeria, respectively, found that factors associated with the use of herbal medicine by patients with T2DM were: low socioeconomic level, rural residence, and age at onset of diabetes [9,10]. In a study carried out in 2021 in an Ethiopian government hospital on 395 patients with T2DM, more than half of the patients used herbal medicine, in particular 58.5%. The factors favoring its use were: gender, place of residence, level of education, duration of the disease, and presence of complications related to diabetes [11].

Diabetes management is a real public health problem in Cameroon. The prevalence of diabetes is estimated at 5.5% in 2021 and could reach 8-40% in certain populations, such as groups of prisoners, as found in a 2020 study in Yaoundé Central Prison, or patients with stroke or chronic kidney disease [12-14]. In the West region of Cameroon, the use of herbal medicine for the treatment of many diseases, particularly diabetes, is part of an ancient tradition. Although there is little evidence of factors that favor its use in T2D patients, there is no data on the prevalence of phytotherapy consumption and its determinants. Based on this observation, we were motivated to conduct this study, the main objective of which was to determine the prevalence of phytotherapy consumption and identify the determinants, in order to improve the quality of health care for patients living with diabetes. in Cameroon.

## Methods

**Study design:** a cross-sectional study was conducted on participants living with type 2 in the Dschang Health District (DHD) from January to May 2022.

**Study setting and population:** this study was carried out in the Dschang Health District. The latter follows the geographical contours of the Menoua subdivision in the Western Region of Cameroon. It covers a surface area of 1060 Km^2^ and is inhabited by around 240,000 people. Four of the six administrative precincts of Menoua are included in this health district (namely: Dschang; Fokoué; Nkong-ni; Fongo Tongo and the Fondonera group) (Dschang Health District) [15]. Many health facilities are present in this district, the main ones being Dschang Regional Hospital Annex (previously known as Dschang District Hospital), Saint Vincent de Paul Hospital, and Batsingla Hospital. These three health facilities each have an internal medicine department and a unit for monitoring people living with diabetes. The people living with diabetes are also organized in association at the level of the community.

**Participants:** the study population was patients living with type 2 diabetes followed in these three health facilities and/or living in the Dschang Health District. Patients with diabetes were defined as patients with a documented history of diabetes or meeting the American Diabetes Association 2022 criteria for diabetes who were considered diabetic, whether or not taking antidiabetic medication [16]. Participants were recruited at the Dschang District Hospital and in the community (homes, workplaces, and health centers). All participants were given clear information about the study and their participation. All participants with type 2 diabetes who had lived in the Dschang Health District for more than one year and who agreed to participate were included in the study. All pregnant participants with T2D and those who withdrew their consent during the study were excluded.

**Variables:** the collected variables included sociodemographic data, history of diabetes, treatment of diabetes, phytotherapy consumption, and its motivation. Phytotherapy (herbal medicine) was defined as a therapeutic method that uses plants or their extracts in the treatment of disease. Conventional medicine, on the other hand, was defined as the use of medicines purchased from a pharmacy. Drugs bought over the counter (street) were also considered as conventional medicine. Phytotherapy consumption was the outcome variable. Independent variables included the age, sex, educational level, religion, and the motivation for phytotherapy consumption. We considered possible confounders in our analysis.

**Data resources and management**

**Data collection tool:** data were collected using a pre-tested, standardized, anonymized questionnaire designed for this purpose. The information collected was then stored in a computer database. This structured questionnaire included all the above-listed variables.

**Data collection:** data were collected from the patient alone or, if necessary, in the presence of an interpreter with the patient's consent to facilitate communication. Patients were provided with an information leaflet explaining the aims of the study, and additional explanations were given if requested. Information was collected only after a positive response from the patient, who was given a consent form to sign. Information about the management of their disease was recorded. The study variables were socio-demographic data: age, sex, marital status, occupation, monthly income, region of origin, place of residence, religion, and level of education). Information on diabetes knowledge and practices was also collected: duration of diabetes, possession of a blood glucose meter, self-monitoring of blood glucose and type of treatment used. Data on perceptions of care (modern medicine, traditional medicine: exclusive use of one approach, combination of the two, therapeutic preference, elements of choice: cost; effectiveness; accessibility; cultural values) were also recorded. The type of treatment used and reason for using it were also registered.

**Sample size:** the sample size was calculated using Lorenz´s formula (Stat Calc of EPI Info Software). Using the national prevalence of 5.5% in Cameroon (according to IDF 2021 data), with an 80% power to detect significant associations or differences, and a 5% accepted margin of error. This gave us a minimum sample size of 80 patients using a margin of error of 5% (standard value of 0.05) [16].


$$N=\frac{\left(p\right)\left(1-p\right){\left(z\right)}^{2}}{d^{2}}$$

Where N=sample size, p=prevalence, z=constant, and d=level of precision. The health areas in which the recruitment was to take place were randomly drawn and 6 of them were selected. Participants were selected consecutively.

**Statistical analysis:** the data collected on the survey sheets were processed on Excel® 2016 (Microsoft Corp., Washington, USA). This data was analyzed using Statistical Package for the Social Sciences® (SPSS) version 23.0 (Chicago, IL, USA). Qualitative variables were represented in frequencies and percentages by bar and sector tables and diagrams and quantitative variables were expressed by means and standard deviations. Chi-squared tests and logistic regressions allowed us to investigate the factors associated with the use of herbal medicine in DT2 patients. The significance threshold was defined as p less than 5% with a 95% confidence interval (CI).

**Ethics approval and consent to participate:** this work was approved by the Institutional Council of the Faculty of Medicine and Pharmaceutical Sciences, University of Douala, Cameroon (Ethical Clearance 2959 CEI-Udo/02/2022/T from the Institutional Ethics Committee of the University of Douala for Human Health Research) and Obtaining Research Authorization from the Head of the DSD, the Director of the Dschang District Hospital (HDD). We conducted this study in strict compliance with the fundamental principles of scientific research in medicine. Patients were free to participate in the study without external constraints. We obtained an informed and signed consent form from each participant.

## Results

**Figure 1:**
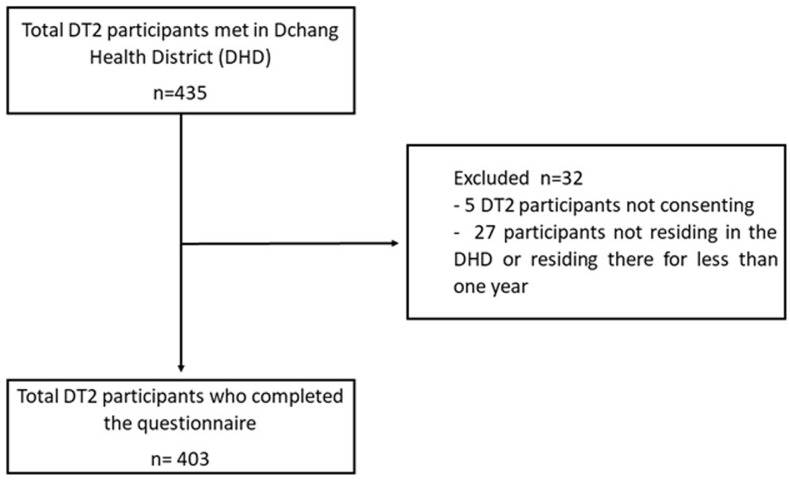
study population flowchart of participants recruited in the Dschang Health District

**Sociodemographic analysis:** we recruited 403 (249 women) T2D patients with a median age of 63 ± 14.86 years. Most of them were unemployed and had a monthly income lower than 50,000 FCFA (76.29 USD). Figure 1 presents the patient´s flow chart.

**Figure 2:**
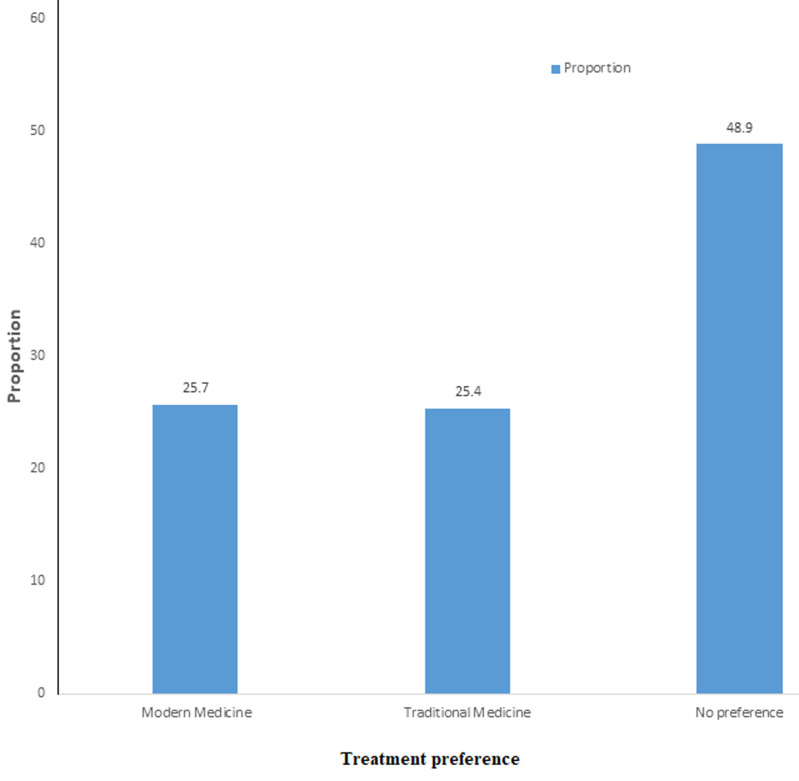
distribution of participants with type 2 diabetes recruited in the Dschang Health District by treatment preference

**Frequency of phytotherapy products use:** among our participants, 240 (59.55%) used phytotherapy, whether associated with conventional treatment (168 (41.69%) participants) or not (72 (17.86%) participants). The reasons for use declared by participants were: low cost (27.8%), ease of accessibility (36.1%), and belief in efficacy (36.1%). Most patients used both treatments because they thought the combination was more effective (69.1%). In addition, just over a quarter (25.7%) of participants preferred conventional treatment alone, while another quarter (24.4%) preferred herbal medicine alone, and almost half (48.9%) had no preference (Figure 2).

**Bivariate analysis:** in bivariable analysis, we observed an association between level of education (p=0.003), socioeconomic level (p <0.001), place of residence (p=0.003), duration of diabetes (p=0.007) and use of phytotherapy (Table 1).

**Table 1:** factors associated with the use of phytotherapy in univariate analysis among participants recruited in the Dschang Health District

Variables	Items	Type of treatment				Total (%)	P-value
		No treatment (%)	Both (%)	Conventional medicine (%)	Traditional medicine (%)		
Occupation	Self-employment	28 (20.9)	57 (42.5)	24 (17.9)	25 (18.7)	134 (100)	0.016
	Unemployed	46 (27.7)	68 (41.0)	18 (10.8)	34 (20.5)	166 (100)	
	Withdrawal	6 (13.0)	20 (43.5)	11 (23.9)	9 (19.6)	46 (100)	
	Salaried	12 (21.1)	23 (40.4)	18 (31.6)	4 (7.0)	57 (100)	
Monthly income	100000-150000	7 (21.9)	14 (43.8)	8 (25.0)	3 (9.4)	32 (100)	<0.001
	25000-50000	36 (21.7)	70 (42.2)	25 (15.1)	35 (21.1)	166 (100)	
	51000-100000	10 (13.0)	36 (46.8)	19 (24.7)	12 (15.6)	77 (100)	
	Less than 25000	37 (37.8)	34 (34.7)	6 (6.1)	21 (21.4)	98 (100)	
	No fixed income	0 (0.0)	4 (57.1)	2 (28.6)	1 (14.3)	7 (100)	
	≥150000	2 (8.7)	10 (43.5)	11 (47.8)	0 (0.0)	23 (100)	
Level of education	Not in school	33 (27.5)	48 (40.0)	12 (10.0)	27 (22.5)	120 (100)	0.003
	Primary	36 (20.1)	76 (42.5)	29 (16.2)	38 (21.2)	179 (100)	
	Secondary	21 (23.3)	36 (40.0)	26 (28.9)	7 (7.8)	90 (100)	
	Superior	2 (14.3)	8 (57.1)	4 (28.6)	0 (0.0)	14 (100)	
Place of residence	Rural	50 (28.1)	56 (31.5)	24 (13.5)	48 (27.0)	178 (100)	<0.001
	Semi-urban	31 (19.1)	76 (46.9)	34 (21.0)	21 (13.0)	162 (100)	
	Urban area	11 (17.5)	36 (57.1)	13 (20.6)	3 (4.8)	63 (100)	
Duration of the diabetes	1-3 years	8 (15.1)	25 (47.2)	15 (283)	5 (9.4)	53 (100)	0.007
	3-5 years	30 (27.8)	43 (39.8)	16 (14.8)	19 (17.6)	108 (100)	
	5-10 years	28 (23.3)	41 (34.2)	22 (18.3)	29 (24.2)	120 (100)	
	6-12 months	2 (6.5)	15 (48.4)	11 (35.5)	3 (9.7)	31 (100)	
	≥10 years	24 (26.7)	43 (47.8)	7 (7.8)	16 (17.8)	90 (100)	

**Multivariate analysis:** in multivariate analysis, only the age group between (51 and 60 years old) (OR: 0.50,95% CI 0.298 - 0.8521; p=0.01) was associated with the use of herbal medicine (Table 2). In addition, females were 1.5 times more likely to use phytotherapy and unemployed patients were 1.9 times more likely to use phytotherapy even though they were not associated with phytotherapy consumption. Similarly, having a monthly income of fewer than 25,000 FCFA (38.14 USD) increased the risk of using phytotherapy 2.6 times (Table 2).

**Table 2:** factors associated with the use of phytotherapy in multivariate analysis among participants recruited in the Dschang Health District

Variables	Items	Odd ratio (OR)	95% confidence interval for OR		P-value
			Lower bound	Upper bound	
Sex	Female	1.565	0.976	2.509	0.063
	Male	NA	NA	NA	NA
Age	21-30	NA	NA	NA	
	31-40	0.293	0.056	1.533	0.146
	41-50	0.554	0.267	1.149	0.113
	51-60	0.504	0.298	0.851	0.010
	61-80	NA	NA	NA.	NA.
Religion	Animist	1.223	0.561	2.664	0.613
	Christian	1.463	0.716	2.991	0.297
	Muslim	2.474	0.623	9.827	0.198
	No religious obedience	NA	NA	NA	NA
Profession	Self-employment	1.859	0.898	3.850	0.095
	Unemployed	1.967	0.868	4.458	0.105
	Retired person	1.997	0.773	5.157	0.153
	Salaried employee	NA	NA	NA	NA
Monthly income	100.000-150.000	1.105	0.346	3.533	0.866
	25.000-50.000	1.028	0.359	2.949	0.958
	51000-100000	1.651	0.584	4.668	0.344
	150000-500.000	0.607	0.185	1.994	0.410
	0-25.000	2.151	0.310	14.901	0.438
	500.000-1.000.000	NA	NA	NA	NA
Level of education	Out of school	0.936	0.224	3.907	0.928
	Primary	0.851	0.216	3.360	0.818
	Secondary	0.443	0.114	1.721	0.239
	Superior	NA	NA	NA	NA
Place of Residence	Rural	0.739	0.373	1.465	0.387
	Semi-urban	0.886	0.462	1.699	0.716
	Urban area	NA	NA	NA	NA
Diabetes duration	1 - 3 years	0.910	0.397	2.089	0.825
	3 - 5 years	0.854	0.427	1.709	0.656
	5 - 10 years	0.844	0.449	1.588	0.599
	1/2 - 1 years	0.775	0.293	2.049	0.607
	10 - 40 years	NA	NA	NA	NA

NA: not applicable

## Discussion

The main objective of this study was to determine the prevalence and risk factors of phytotherapy use among patients with type 2 diabetes in a sub-Saharan Africa setting, the Dschang Health District in Cameroon. This study represents one of the few studies on the use of phytotherapy among T2D patients in Cameroon and showed frequently used phytotherapy as an antidiabetic remedy. Factors associated with its use were age between 51 and 60 years, low education level, low socioeconomic level, and medium duration of diabetes.

This cross-sectional study should be interpreted in light of some limitations. As we carried out this study in only one health district with urban and rural populations, findings can not be extrapolated to others. The other limitations were the size of the population (small), compared to other studies on the same topic, and the fact that much information relies on patient declarations. These could be inexact due to memory bias or lying. There is a great interest in the use, efficacy, and tolerability of phytotherapy in the management of T2D in Western and Asian countries. Recent studies in East and North African countries have shown a huge use of phytotherapy for the treatment of T2D with mixed results. To the best of our knowledge, there are no data on its prevalence and risk factors in Central Africa, particularly in Cameroon, when used to treat diabetes.

Of the participants included, the majority were female (61.8%) with a mean age of 63.77 ± 14.86 years. These findings corroborate a study by Alioune Camara *et al*. in 2014 and Simeni Njonnou *et al*. in 2020, where diabetes patients were predominantly female (61% and 66.2%, respectively) [17,18]. Doukani *et al*. also in Algeria in 2020, found that 58.75% of patients with diabetes were female [10]. An older population was also found by Youb *et al*. Selihi *et al*. and Hamza *et al*. [9,19,20]. This could be explained by the fact that insulin resistance increases with age and, in our context, women visit health facilities more often than men and their life expectancy is higher than that of men [14].

Almost a third (29.8%) of participants have never attended school while 44.4% had completed primary education. These results are similar to those found by Hamza *et al*. in 2011 in Algeria and Selihi *et al*. in Morocco where the majority of the participants of the patients were illiterate [9,20]. These findings are different from that of Doukani *et al*. in Algeria where there was no real difference among the study population concerning the educational level, as well as in a Canadian study [3,10]. This difference could be explained by the fact that the majority of the participants in our study came from rural areas and were of advanced age.

In this study, the percentage of T2D participants who used phytotherapy for the control or treatment of diabetes was 59.55%. This is similar to the results found in 2021 by Wahiba *et al*. (60%), and by Kasole *et al*. (67.2%) in Tanzania [21,22]. On the contrary, it is far superior to the results found in a Canadian study conducted by Grossman *et al*. in 2018 where the prevalence was 44% and also above 49% found in the literature in a study conducted by Hamza *et al*. in Algeria [3,20]. Our results could be explained by the fact that in our context, cultural values promote herbal medicine and access to care remains a real public health problem.

Participants aged between (60-80 years) used herbal the most (114 patients) while patients aged between (30-40 years) (4 patients) used less. This result is similar to the results found in the literature by Selihi *et al*. in Morocco where the majority of phytotherapy consumers (46.5%) were above the age of 60 [9]. However, these findings differ from the results found by Kasole *et al*. where T2D participants consuming phytotherapy were aged between (41 - 60 years) (59.8%) [22]. This could be explained by the fact that our study took into account patients living both in rural areas (who are mostly elderly) and in urban areas. Their attachment to traditional medicine could be explained by the fact that most patients in these age groups are much more attached to customs and traditions.

The prevalence of phytotherapy consumption among female T2D participants taking treatment was 82%. Similarly, the female gender had 1.5 times the risk of phytotherapy consumption compared to the male gender. Similar results were found in 2016 by Adouane *et al*. Doukani *et al*. and by Kasole *et al*. [10,22,23]. Hamdoun *et al*. and Errajraji *et al*. also found a significant association between female gender and phytotherapy consumption [24,25]. This result could be explained by the relatively high frequency of illiteracy of women in rural areas in our context, their vigilance for the balance of the disease, the ease of transmission of information between them, the lowest cost, the availability of traditional medicines, and women´s greater attachment to all that is traditional [26,27].

The majority of participants who used phytotherapy in our study were unemployed. Moreover, being unemployed increased 1.9 times the risk of using phytotherapy compared to the participants who were employed. In addition, in our study, there was a statistically significant association between occupation and phytotherapy consumption. The use of plants for the treatment of diabetes was also associated with low educational level and low socioeconomic level. These results are similar to those found by Kasole *et al*. Hamdoun *et al*. Hamza *et al*. and Errajraji *et al*. where educational level and socioeconomic level were associated with the use of herbal medicine [20,22,24]. These studies corroborate the work by Selihi *et al*. on herbal medicine and degenerative complications of diabetes where the level of education, the socioeconomic level were significantly associated with the use of herbal medicine [9]. However, Doukani *et al*. found that the level of education had no significant influence on the use of phytotherapy to treat diabetes and the most represented socioeconomic level was the middle class [10]. These different elements favoring the use of plants, since in our context, care is generally the responsibility of patients or their families, thus limiting access to the conventional health system [27].

In this study, the majority of participants surveyed lived in rural areas (44.2%) with the majority of participants using phytotherapy. By the same, we had an association between residence location and the use of phytotherapy. This can be due to the fact they were very far away or did not have easy access to the medical centers supporting patients with diabetes. These results corroborate those found by Selihi *et al*. Hanae *et al*. and Doukari *et al*. where the majority of patients using herbal medicine lived in rural areas [9,10,28]. Some medical centers with general practitioners do not fully manage the disease due to insufficient technical support (lack of refrigerator for storing injectable antidiabetics and unavailability of all classes of oral antidiabetics) therefore use only available oral antidiabetics.

We found that participants whose duration in diabetes was between (5-10 years) used more herbal medicine (58.8%) compared to those whose duration was less than one year. In our study, the disease duration was associated with the use of herbal medicine. This is in line with the findings of Selihi *et al*. and Kasole *et al*. [9,22]. In addition, the use of medicinal plants can be explained by the fact that participants faced “relative inefficiency” of conventional treatment, as it fails to cure the disease and, if not well adapted, also fails to control glycemia and prevent chronic complications. [7].

Belief in efficacy, accessibility, and low cost were the main reasons driving phytotherapy consumption. This was similar to the results found in most studies in Africa although an influence of culture or tradition has been reported. [8-10,20,21,24]. Particular work should be done to bring treatment centers closer to patients and improve their technical platform.

## Conclusion

Despite its limitations, this study which is to the best of our knowledge the first of its kind in Cameroon on T2D showed a huge consumption of phytotherapy in the Dschang Health District. Determinants of phytotherapy consumption in this study population included old age, residence in a rural area, and low income. The main reasons for consuming phytotherapy were the belief in efficacy, its accessibility, and its low cost. Important work should be done by stakeholders and medical staff to enhance patients´ education, and the technical level of treatment centers and bring them closer to patients. There is a need to evaluate its efficacy in treating diabetes and its adverse effects.

### What is known about this topic

- *Phytotherapy is widely used in sub-Saharan Africa for treating several diseases;*
- *Phytotherapy was used in many forms (barks, powder, decoctions) to treat diabetes*.

### What this study adds

- *Almost 6 people over 10 with T2D are using phytotherapy;*
- *The main reasons for using it were: low cost (27.8%); accessibility (36.1%); and belief in efficacy (36.1%);*
- *In multivariate analysis, only the age group between (51 and 60 years old) was associated with the use of herbal medicine*.
